# Supplementary material for: Leveraging human genetic variation to therapeutically target hundreds of genes with dominant & dispensable disease alleles
Source: medRxiv. 2026 Mar 27:2026.03.26.26349431. Preprint. [Version 1] doi: 10.64898/2026.03.26.26349431 (PMC13042096; doi:10.64898/2026.03.26.26349431)
Supplement: Supplement 5 [file NIHPP2026.03.26.26349431v1-supplement-5.pdf]

medRxiv preprint doi: https://doi.org/10.64898/2026.03.26.26349431; this version posted March 27, 2026. The copyright holder for this preprint (which was not certified by peer review) is the author/funder, who has granted medRxiv a license to display the preprint in perpetuity.
It is made available under a CC-BY 4.0 International license .

Supplementary Figures

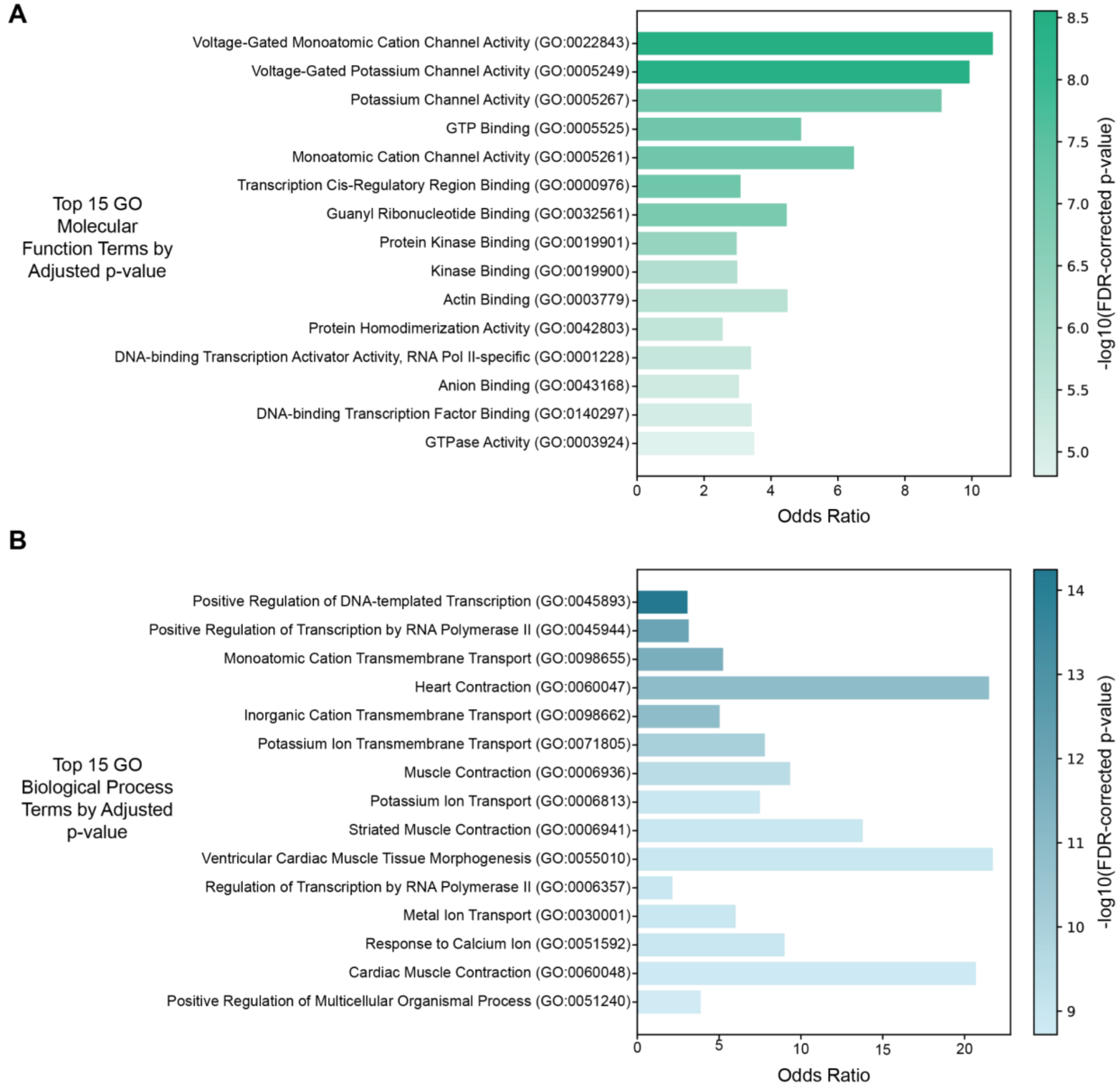

Supplementary Figure 1. D&D genes exhibit enrichments across many molecular functions and biological processes. (A) Top 15 GO molecular function enrichments among D&D genes. Most prominent terms are related to ion channel activity (consistent with nervous system enrichments in the human phenotype ontology (HPO) catalog), protein and DNA binding, and kinase activity. (B) Top 15 GO biological process enrichments for D&D genes. Most prominent terms are related to ion channel activity, DNA binding, and heart and muscle contraction response, consistent with cardiac system enrichments among HPO terms for the genes. All pictured terms are significant after FDR correction.

medRxiv preprint doi: https://doi.org/10.64898/2026.03.26.26349431; this version posted March 27, 2026. The copyright holder for this preprint (which was not certified by peer review) is the author/funder, who has granted medRxiv a license to display the preprint in perpetuity.
It is made available under a CC-BY 4.0 International license .

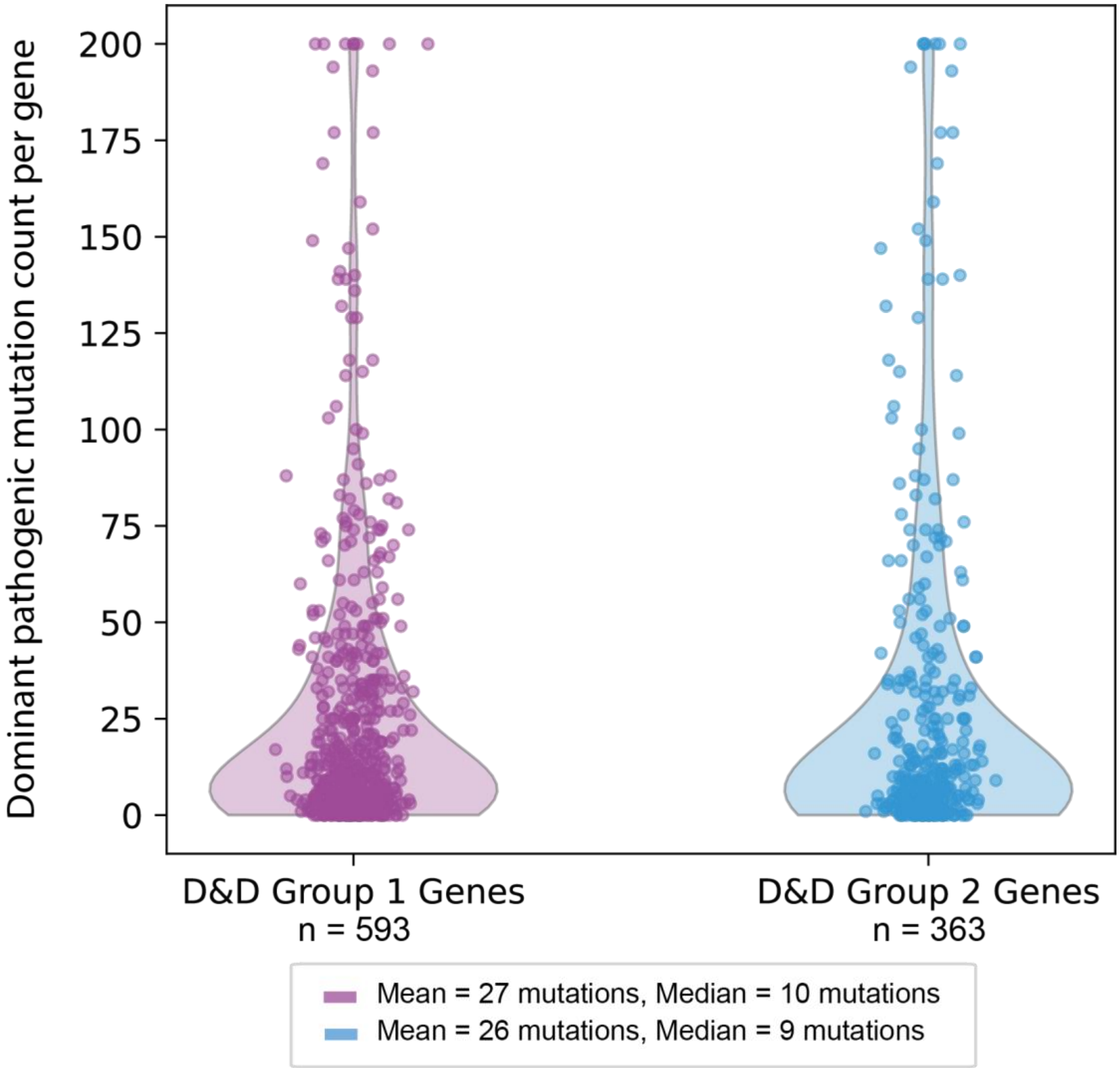

Supplementary Figure 2. With high pathogenic mutation count, disease mutation-agnostic editing could provide dramatic therapeutic benefit across D&D Group 1 and Group 2 genes. Numbers of dominant pathogenic mutations among D&D Group 1 and Group 2 genes are shown. With high mean and median numbers of disease-causing mutations, mutation-agnostic editing could reach more patients and reduce regulatory burdens for large numbers of therapies. Note that D&D Group 2 genes are a subset of D&D Group 1 genes.

medRxiv preprint doi: https://doi.org/10.64898/2026.03.26.26349431; this version posted March 27, 2026. The copyright holder for this preprint (which was not certified by peer review) is the author/funder, who has granted medRxiv a license to display the preprint in perpetuity.
It is made available under a CC-BY 4.0 International license .

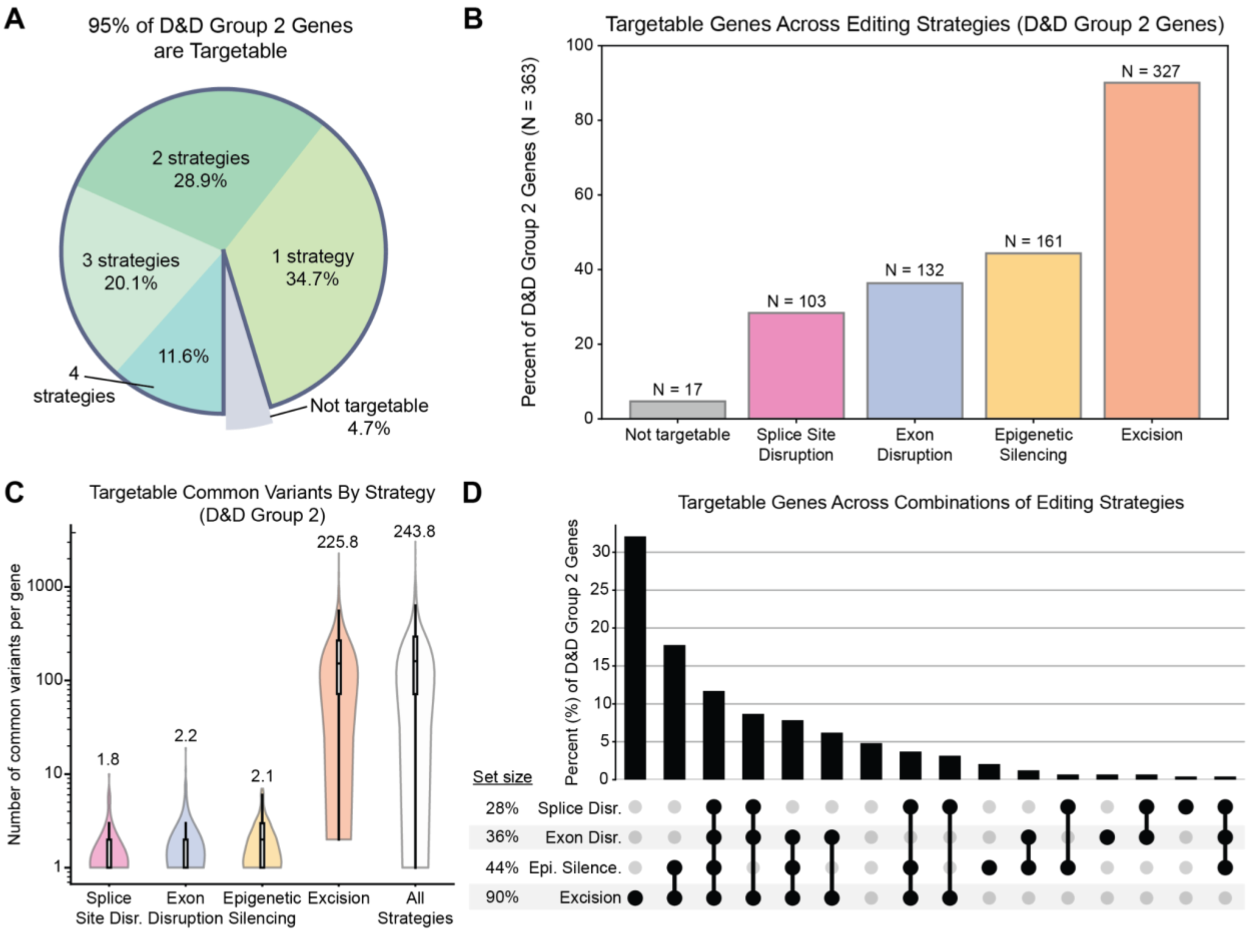

Supplementary Figure 3. Common-variant-mediated gene editing strategies enable targeting of >95% of D&D Group 2 genes. (A) Over 95% of D&D Group 2 genes were targetable with at least one of the four editing strategies, and over half of the D&D genes (54%) were targetable by more than one strategy. (B) Percentage of D&D Group 2 genes targetable by each editing strategy. Based on common variant patterns, excision could target the most genes (90%), followed by epigenetic silencing (44% of genes), exon disruption (36% of genes), and splice site disruption (27% of genes). (C) Numbers of common variants for each D&D Group 2 gene targetable by each editing strategy, indicating many opportunities to target each gene. Numbers above violins are mean values of each distribution. (D) D&D genes are often targetable by multiple strategies, providing therapeutic versatility.

medRxiv preprint doi: https://doi.org/10.64898/2026.03.26.26349431; this version posted March 27, 2026. The copyright holder for this preprint (which was not certified by peer review) is the author/funder, who has granted medRxiv a license to display the preprint in perpetuity.
It is made available under a CC-BY 4.0 International license .

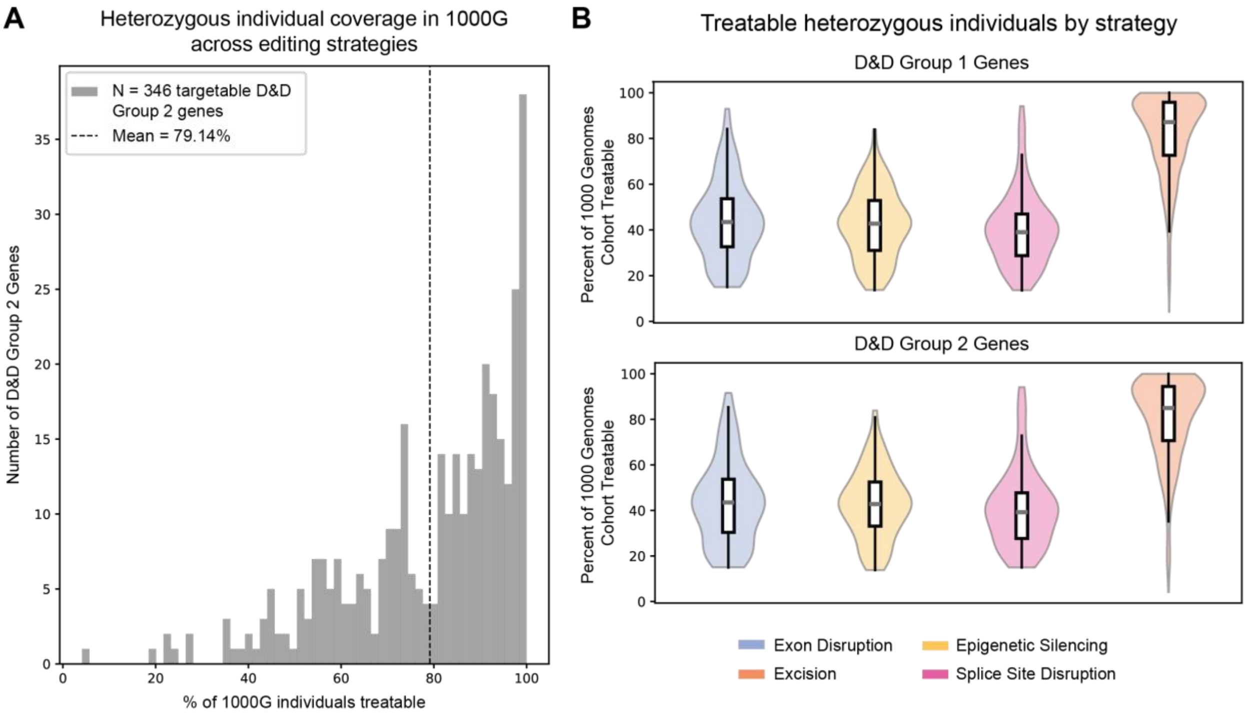

Supplementary Figure 4. Over 75% of people are therapeutically targetable for D&D Group 2 genes. (A) Percent of total 1000 Genomes cohort that is heterozygous at at least one common variant site for each D&D Group 2 gene, combining unique people across each gene editing strategy. (B) Percent of total 1000 Genomes cohort treatable per gene, broken down by editing strategy for D&D Group 1 Genes (top) and D&D Group 2 Genes (bottom). 1000G = 1000 Genomes.

medRxiv preprint doi: https://doi.org/10.64898/2026.03.26.26349431; this version posted March 27, 2026. The copyright holder for this preprint (which was not certified by peer review) is the author/funder, who has granted medRxiv a license to display the preprint in perpetuity.
It is made available under a CC-BY 4.0 International license .

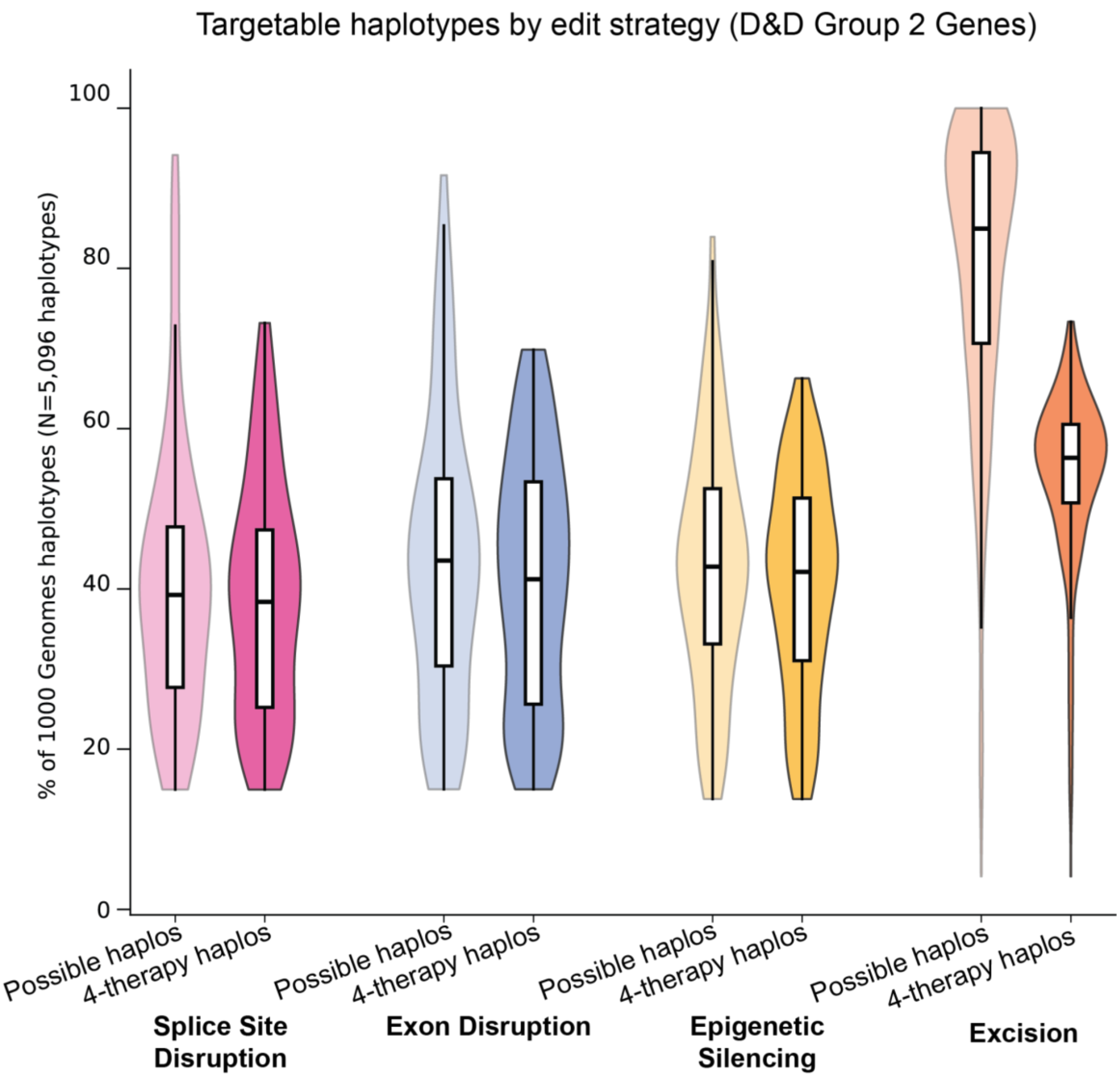

Supplementary Figure 5. Across a majority of editing strategies, only 4 therapies are required to target a majority of possible haplotypes in the population for D&D Group 2 Genes. Percent of haplotypes out of the total number of haplotypes in the 1000 Genomes cohort that are either targetable at all (by virtue of harboring a heterozygous common variant allele), denoted as "Possible haplos", or targetable by the first 4 selected therapies, denoted as "4-therapy haplos". For splice site disruption, exon disruption, and epigenetic silencing, 4 therapies could target nearly all possible haplotypes, with less than a 3% drop off between Possible haplos and 4-therapy haplos. Furthermore, the excision strategy could target 54% of haplotypes on average across genes with only 4 therapies, capturing a large proportion of cohort haplotypes.

medRxiv preprint doi: https://doi.org/10.64898/2026.03.26.26349431; this version posted March 27, 2026. The copyright holder for this preprint (which was not certified by peer review) is the author/funder, who has granted medRxiv a license to display the preprint in perpetuity.
It is made available under a CC-BY 4.0 International license .

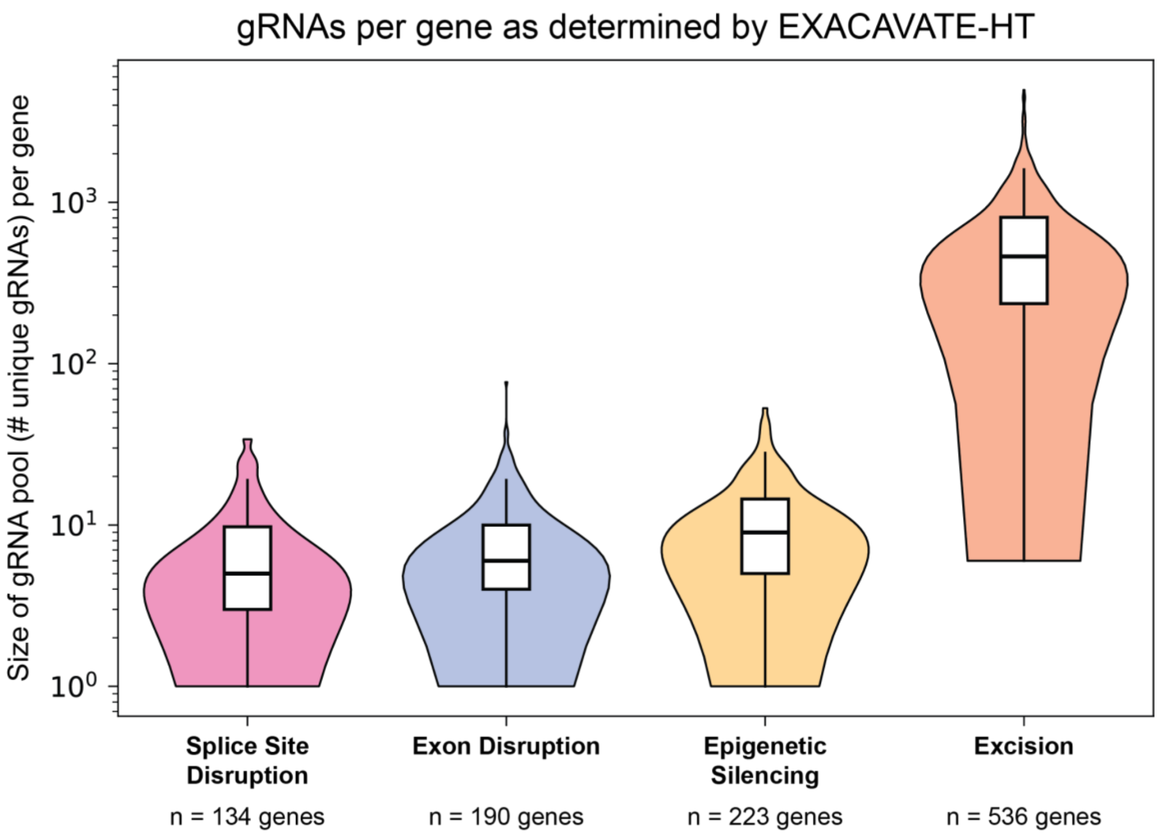

Supplementary Figure 6. gRNA pools per D&D gene. Number of unique gRNAs available per targetable D&D gene, determined by EXCAVATE-HT. gRNAs were generated by assessing CRISPR/SpCas9 targetability of each common variant and NGG PAM site proximity. Note that numbers of genes targetable by each editing strategy may be lower than in Figure 3C due to some genes not having any common variants targetable by CRISPR/SpCas9.

medRxiv preprint doi: https://doi.org/10.64898/2026.03.26.26349431; this version posted March 27, 2026. The copyright holder for this preprint (which was not certified by peer review) is the author/funder, who has granted medRxiv a license to display the preprint in perpetuity.
It is made available under a CC-BY 4.0 International license .

A

Session Link: https://genome.ucsc.edu/s/gramey02/MYH7_example_session

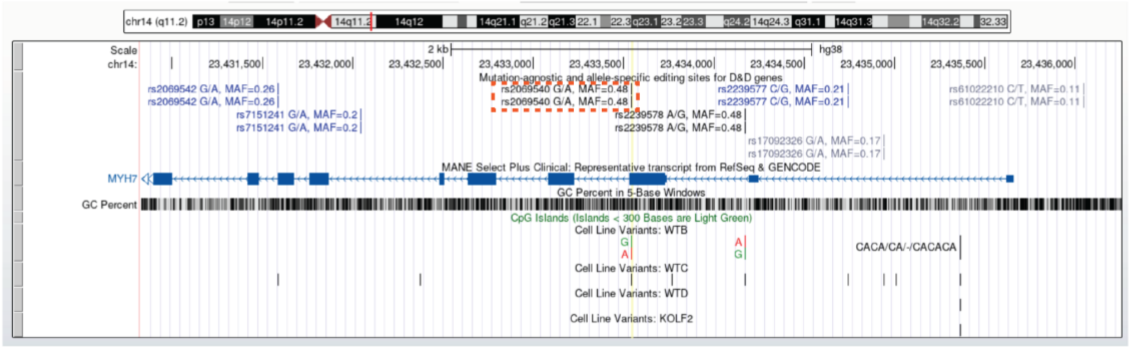

B

D&D gene editing targets (Item Details)

Item: rs2069540 G/A, MAF=0.48
Score: 0
Position: chr14:23433544-23433544
Band: 14q11.2
Genomic Size: 1
View DNA for this feature (hg38/Human)

**Description**	A therapeutically editable common variant for the DnD gene MYH7
**Ref/Alt allele**	G/A
**Allele frequency (AF)**	0.52
**Minor allele frequency (MAF)**	0.48
**Global genotype frequencies in 1000 Genomes**	Heterozygous (GA) 47% (1220/2548), Homozygous ref (GG) 24% (613/2548), Homozygous alt (AA) 28% (715/2548)
**AFR genotype frequencies (1000 Genomes)**	Allele frequency = 0.64, Heterozygous (GA) 46% (315/671), Homozygous ref (GG) 12% (84/671), Homozygous alt (AA) 40% (272/671)
**EUR genotype frequencies (1000 Genomes)**	Allele frequency = 0.48, Heterozygous (GA) 45% (239/521), Homozygous ref (GG) 28% (149/521), Homozygous alt (AA) 25% (133/521)
**AMR genotype frequencies (1000 Genomes)**	Allele frequency = 0.41, Heterozygous (GA) 51% (179/348), Homozygous ref (GG) 33% (117/348), Homozygous alt (AA) 14% (52/348)
**EAS genotype frequencies (1000 Genomes)**	Allele frequency = 0.41, Heterozygous (GA) 47% (245/515), Homozygous ref (GG) 34% (180/515), Homozygous alt (AA) 17% (90/515)
**SAS genotype frequencies (1000**	Allele frequency = 0.59, Heterozygous (GA) 48% (241/492), Homozygous ref (GG) 16% (83/492), Homozygous alt (AA) 34% (168/492)

C

**Targetable by the following CRISPR editing strategies**	excision, exon disruption, splice site disruption
**Five closest excision partners, if any**	23382191,23382673,23383442,23384420,23385115
**CRISPR/SpCas9 Targetable?**	Yes
**+/- 25 bp flanking variant**	GCCTGACACCCACCTTGCCATACTC_G_GTCTCGGCAGTGACTTTGCCACCCT
**Cas/gRNA generation tools**	CRISPOR - https://crispor.gi.ucsc.edu/, CRISPick - https://portals.broadinstitute.org/gppx/crispick/public

Supplementary Figure 7. Mutation-agnostic gene editing candidates TrackHub on UCSC Genome Browser. (A) Snapshot of the provided genome browser resource for the gene *MYH7*, with session link that users can click on to be brought to the browser directly (https://genome.ucsc.edu/s/gramey02/MYH7_example_session). Common variants targetable by mutation-agnostic editing strategies are shown. Note that variants in this window show up twice because the nearby *MYH6* gene is also targetable by excision at some of these sites. Orange dotted box is the common variant highlighted in B & C. (B & C) Snapshots of the clickable inset information provided for each common variant on the track, including subpopulation-specific allele frequencies (B), editing strategy information (C), and flanking sequences (C). Additional tools for gRNA identification are also listed (C).

medRxiv preprint doi: https://doi.org/10.64898/2026.03.26.26349431; this version posted March 27, 2026. The copyright holder for this preprint (which was not certified by peer review) is the author/funder, who has granted medRxiv a license to display the preprint in perpetuity.
It is made available under a CC-BY 4.0 International license .

Supplementary Tables

Supplementary Table 1. HPO and GO Term Enrichments.

Supplementary Table 2. D&D gene targetability by edit strategy.

Supplementary Table 3. Main figure data.

Supplementary Table 4. Manual organ system annotations for missing HPO genes.
